# Filling-in of the foveal rod scotoma and confidence preference for central vision under mesopic viewing conditions

**DOI:** 10.1016/j.visres.2026.108794

**Published:** 2026-03-02

**Authors:** Hui Men, Alexander C. Schütz

**Affiliations:** a AG Sensomotorisches Lernen, Philipps-Universität Marburg, Marburg, Germany; b Center for Mind, Brain and Behaviour, Marburg, Gießen, Darmstadt, Germany

**Keywords:** Photopic vision, Mesopic vision, Scotopic vision, Cones, Rods, Filling-in, Confidence

## Abstract

Different photoreceptors support vision depending on ambient light levels: cones mediate photopic vision at high light levels, while rods mediate scotopic vision at low light levels. In mesopic vision at intermediate light levels, both rods and cones are active. Due to the absence of rods in the center of the visual field, the fovea, a foveal scotoma occurs in scotopic vision. Previous research showed that this scotoma can be perceptually filled in using surrounding visual information, and that humans place more trust in such inferred central information than in veridical peripheral input. However, it is unknown whether this phenomenon also occurs in mesopic vision, when cones are still active in the fovea. To this end, we investigated perceptual filling-in of the foveal rod scotoma and confidence judgments under photopic, scotopic, and mesopic conditions. Under mesopic conditions, rods and cones were stimulated independently using a tetrachromatic projector. Participants were asked to discriminate the continuity of two sequentially presented stimuli and to indicate their confidence using a forced-choice task. In foveal rod-isolating conditions, participants could not distinguish between continuous and discontinuous stimuli and showed a bias toward perceiving them as continuous, under both scotopic and mesopic conditions. This indicates that rod-mediated foveal gaps were perceptually filled in by surrounding information, even when cone input was available under the mesopic condition. Furthermore, participants consistently preferred the centrally filled-in information over veridical peripheral information, indicating that the confidence bias toward inferred information within the foveal rod scotoma persists even in mesopic vision.

## 1 Introduction

The human retina contains different types of photoreceptors, rods and cones, which differ in their distribution across the retina (Curcio, Sloan, Kalina, & Hendrickson, 1990) and their sensitivity at different light levels (for reviews see Barbur, Llapashtica, Rider, & Stockman, 2025; Stockman & Sharpe, 2006; Zele & Cao, 2014). Under photopic conditions (bright light), cones are active. Although less sensitive to light, cones enable full trichromatic color vision through three types—L-, M-, and S-cones—each sensitive to different wavelengths corresponding roughly to long (red), medium (green), and short (blue) wavelength light. Cones are densely concentrated in the fovea, the central region of the retina, and gradually decline in density toward the periphery, enabling sharp and detailed vision in bright light at the fovea and low-resolution vision in the periphery (for a review see Strasburger, Rentschler, & Jüttner, 2011). In contrast, under scotopic conditions (dim light), only rods are active. They are highly sensitive to light and allow for vision in very low-light environments, but do not support color perception, resulting in monochromatic (black-and-white) vision. Since rods are densely packed in the peripheral retina but decline sharply toward the fovea, where they are completely absent, this absence leads to a functional foveal scotoma under scotopic conditions. Visual acuity and contrast sensitivity is also significantly reduced in scotopic vision due to the lower spatial resolution of rods. Between photopic and scotopic vision lies mesopic vision, which occurs under intermediate lighting conditions such as during dawn, dusk, or in dimly lit environments. In mesopic vision, both rods and cones are active simultaneously and color perception and visual acuity are reduced compared to photopic vision.

Studies have shown that the scotopic foveal scotoma is perceptually filled in using information from the immediate surround (Gloriani & Schütz, 2019). However, rod signals reach the brain through two primary pathways that vary depending on ambient light levels. Under mesopic conditions, rods not only transmit signals via AII amacrine cells to rod bipolar cells, as they do under scotopic conditions, but also connect directly with cones through gap junctions (for reviews see Bloomfield & Dacheux, 2001; Sharpe & Stockman, 1999). This secondary pathway allows rod signals to enter the cone circuitry more directly, making it unclear whether perceptual filling-in of the rod scotoma persists or is disrupted in mesopic vision.

To make optimal perceptual decisions, the visual system must interpret sensory inputs from different regions of the visual field by utilizing the internal knowledge of its own properties and weighting these inputs according to their estimated reliability (e.g., Ernst & Bülthoff, 2004; Landy, Maloney, Johnston, & Young, 1995; Najemnik & Geisler, 2005). Previous research has shown that humans exhibit a default preference for foveal information not only under photopic vision but also under scotopic vision—even though foveal input in scotopic vision is not veridical but instead filled in (Gloriani & Schütz, 2019). It remains unclear whether this foveal preference persists or is modulated under mesopic viewing conditions.

In this study, we investigated perceptual filling-in of the rod scotoma and confidence preferences under photopic, scotopic, and mesopic viewing conditions. Under the mesopic condition, rods and cones were independently stimulated using a customized tetrachromatic projector. Our findings demonstrate that the absence of rod-mediated input in the fovea is perceptually filled in by signals from the immediate surround—even when cones are simultaneously active and could supply foveal input. Furthermore, we observed a consistent confidence preference across viewing conditions: similar to photopic vision, participants under both scotopic and mesopic conditions tended to place greater trust in centrally inferred (foveal) information than in veridical peripheral input. These results suggest that the preference for inferred information in the foveal rod scotoma persists even under mesopic conditions, indicating a robust and modality-independent bias toward foveal information in perceptual decision-making.

## 2 Methods

### 2.1 Design

To investigate perceptual decision-making and confidence preferences in central and peripheral vision, we employed a confidence forced-choice task (Mamassian, 2020). In each trial, participants were asked to judge the continuity of stimuli presented in two successive intervals. Following each stimulus presentation, they indicated whether the stimulus appeared continuous or discontinuous. At the end of each trial, participants reported for which of the two stimuli they felt more confident about. Compared to traditional confidence ratings, this forced-choice paradigm enables a direct, pairwise comparison of confidence, reducing ambiguity and bias introduced by arbitrary or subjective mappings between internal certainty and an external scale (Mamassian, 2020).

Three viewing conditions—photopic [Pho], scotopic [Sco], and mesopic [Meso]—were tested in separate sessions. In the photopic viewing condition, vision was mediated exclusively by cones [PhoC], while in the scotopic condition, only rods were active [ScoR]. In the mesopic condition, L-cones [MesoC] and rods [MesoR] were independently stimulated, by using the silent substitution method (Bayer, Paulun, Weiss, & Gegenfurtner, 2015; Shapiro, Pokorny, & Smith, 1996). L-cones were selectively isolated under mesopic conditions to achieve higher effective contrast, as stimulating all cone types would have produced lower contrast.

Participants were divided into six groups, with each group experiencing the three viewing conditions in a different order. For each viewing condition, stimuli were presented either in the center (foveal [F]) or in the periphery [P], and the gratings were either continuous [C] or discontinuous [D]. The order of the two stimulus intervals was counterbalanced, resulting in 16 possible display pairings: FD–FD, FD–FC, FC–FD, FC–FC, PD–PD, PD–PC, PC–PD, PC–PC, FD–PD, PD–FD, FD–PC, PC–FD, FC–PD, PD–FC, FC–PC, and PC–FC. Here, FC denotes the foveal-continuous condition, FD the foveal-discontinuous condition, PC the peripheral-continuous condition, and PD the peripheral-discontinuous condition.

This design resulted in 160 trials for each of the photopic and scotopic viewing conditions (16 pairs × 10 repetitions, i.e., 5 repetitions per orientation), and 320 trials for the mesopic condition (2 photoreceptor mediation types × 16 pairs × 10 repetitions, i.e., 5 repetitions per orientation).

### 2.2 Participants

We recruited 27 students from the University of Marburg (20 women, 7 men, mean age = 23 years, range = 18–37 years) to participate in the experiment. All participants had normal or corrected-to-normal vision and provided written informed consent. Before participation, they underwent a color vision assessment using Ishihara plates (Clark, 1924) to confirm normal color perception. They were naïve to the experiment’s purpose and had the option to receive compensation either as course credits or monetarily at a rate of 8€/hour (first 21 participants) and 10€/hour (last 6 participants) for their participation. The experiment was conducted in accordance with the Declaration of Helsinki (1964) and approved by the local ethics committee of the Psychology Department at Marburg University (proposal number 2021–71k).

After exclusions, datasets from 18 participants remained: the datasets of two participants were excluded because their performance in the fovea for rod-isolating stimuli in the mesopic condition (MesoR-Fovea) exceeded 80%, indicating unsuccessful rod–cone isolation. The datasets of two participants were excluded because their performance in the periphery in the scotopic condition (ScoR-Periphery) was less than 70%, indicating a general inability to perform the task. The dataset of one participant was excluded because of a consistent interval bias exceeding the range of 30–70%, indicating a stereotypical response bias in the confidence task. The datasets of four participants were excluded because of invalid eye position data during stimulus presentation in more than 10% of the trials in at least one of the three viewing conditions. Further details on the exclusion criteria are provided in the data analysis section.

### 2.3 Apparatus

Stimuli were presented using Psychtoolbox (Brainard, 1997; Kleiner, Brainard, & Pelli, 2007; Pelli, 1997) in MATLAB R2016b and projected onto a screen measuring 90.70 × 51.00 cm, positioned 106 cm from the participants.

**Fig. 1:**
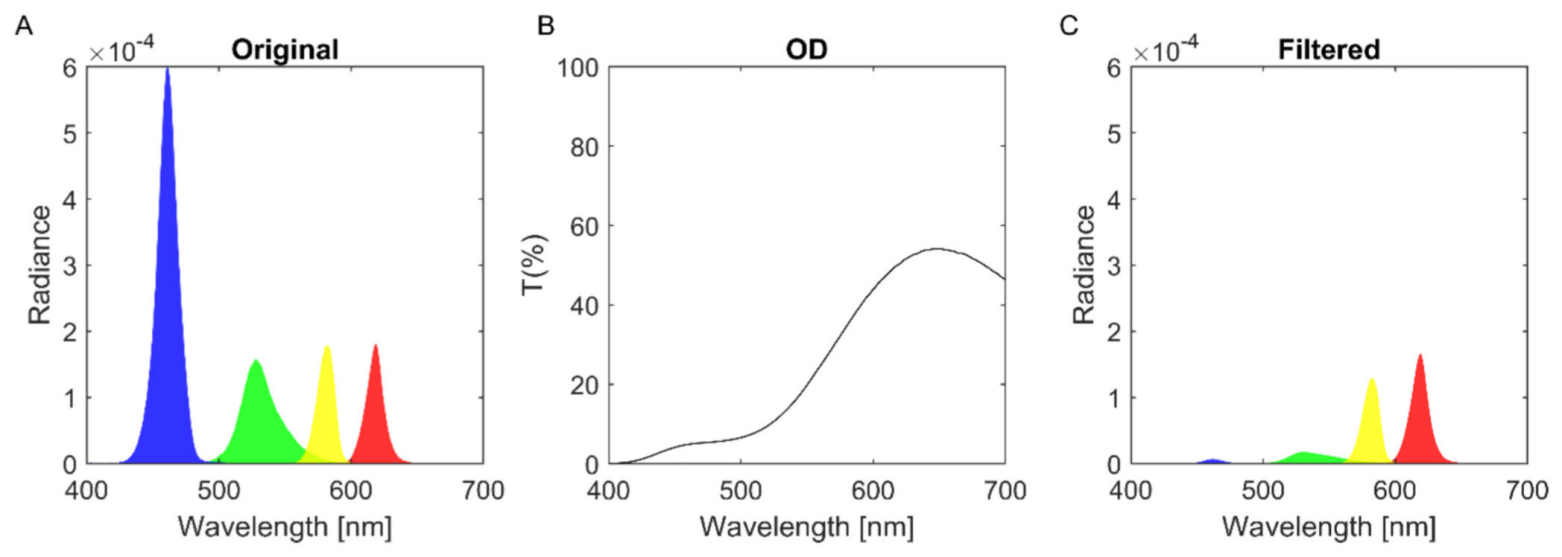
Spectral radiance of the projector’s four channels in the tetrachromatic mode. A) Spectral radiance without filter. B) Transmission spectrum of the yellow glass filter. C) Spectral radiance with the filter.

To achieve independent stimulation of the four photoreceptor types (L-, M-, and S-cones, as well as rods), we employed the silent substitution method based on the CIE 2° standard observer (Shapiro et al., 1996; Stiles & Burch, 1955) using a customized tetrachromatic PROPixx projector (VPixx Technologies Inc., Saint-Bruno, QC, Canada) (Barrionuevo, Schütz, & Gegenfurtner, 2025). This projector features four independent primaries—red, green, blue, and yellow—allowing precise control over photoreceptor activation of all three cone types and of rods (Fig. 1). Silent substitution stimuli were generated using the rod V’(λ) function (CIE Proceedings, 1951) and the 2° cone sensitivities of (Stockman & Sharpe, 2000), which are based on the 10° sensitivities of (Stiles & Burch, 1959).

Viewing conditions were manipulated across three distinct experimental sessions: photopic, scotopic, and mesopic. For the photopic and scotopic conditions, the projector was configured in the standard trichromatic mode, using only red, green, and blue primaries, with a refresh rate of 120 Hz. In the mesopic condition, it operated in the tetrachromatic mode, using red, green, blue, and yellow primaries, with a refresh rate of 100 Hz. The display background was set to a neutral value, defined by RGB values of 128 for red, green, and blue in the trichromatic mode, and by RGBY values of 128 for red, green, blue, and yellow in the tetrachromatic mode. The background appeared gray in the photopic and scotopic conditions and yellowish in the mesopic condition. The yellowish background in the mesopic condition was the consequence of attenuating short wavelengths with a yellow filter to optimize the maximum rod contrast while silencing cones (Fig. 1).

For the photopic condition, the measured luminance values for black, gray, and white pixels were 0.088, 61.300, and 125.700 photopic cd/m^2^, respectively (measured with a SpectroCal MKII spectroradiometer, Cambridge Research Systems Ltd, United Kingdom). To achieve the desired low-luminance levels in the scotopic condition, a set of 17-stop neutral density (ND) glass filters (LEE Filters, Burbank, CA) was mounted in front of the projector, reducing the luminance of black, gray, and white pixels to 6.71 × 10^−7^, 4.68 × 10^−4^, and 9.59 × 10^−4^ photopic cd/m^2^ (nominal values, below the measurement range of the SpectroCal MKII), respectively. For the mesopic condition, yellow glass filters (E-LA140, HOYA CORPORATION OPTICS SECTION Europe Branch, Mönchengladbach, Germany) were installed in front of the projector. Under this condition, the measured luminance values for black, gray, and white pixels were 2.50 × 10^−3^, 1.214, and 2.433 cd/m^2^. The maximum achievable contrast of L-, M-, and S-cones was 0.227, 0.231, and 0.848, respectively, and the maximum contrast for rods was 0.386.

Eye movements of the right eye were recorded at a sampling rate of 1000 Hz using an EyeLink 1000 plus system (SR Research Ltd., ON, Canada), operated via the Eyelink Toolbox (Cornelissen, Peters, & Palmer, 2002). Calibration of the eye tracker was performed prior to the main experiment.

### 2.4 Stimuli

**Fig. 2:**
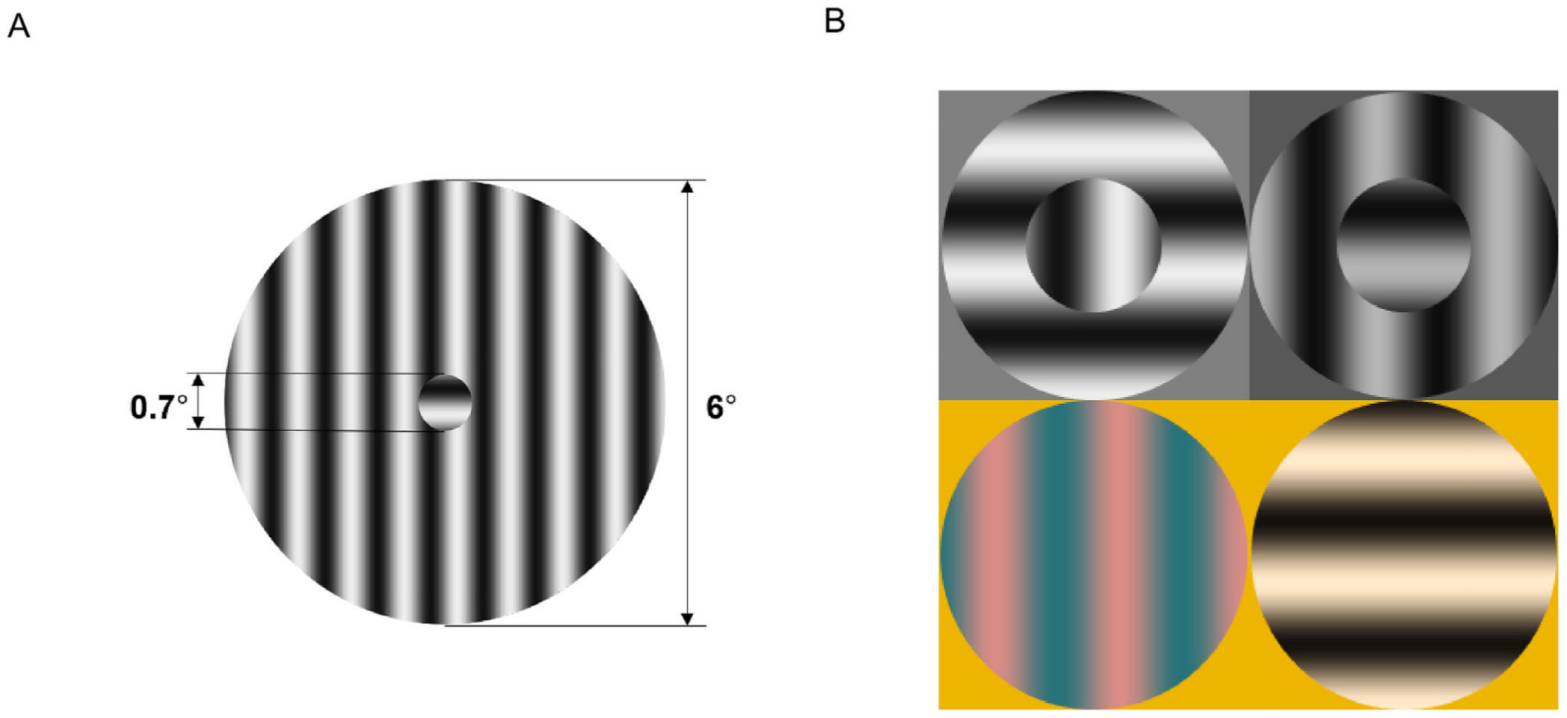
Stimulus conditions. A) A to-scale example of a discontinuous stimulus. B) Four possible stimulus configurations, varying in center-surround continuity (continuous or discontinuous) and orientation (vertical or horizontal), presented under different viewing conditions. Upper row: discontinuous stimuli. Lower row: continuous stimuli. Left column: center vertical aligned. Right column: center horizontal aligned. Upper left: Stimulus mediated by cones under the photopic condition [PhoC]. Upper right: Stimulus mediated by rods under the scotopic condition [ScoR]. Lower left: Stimulus mediated by L-Cones under the mesopic condition [MesoC]. Lower right: Stimulus mediated by rods under the mesopic condition [MesoR]. Stimulus depictions do not match exactly the stimuli in the experiments.

The stimuli consisted of two concentric circles containing sine wave gratings with a spatial frequency of 1.4 cycles per degree (cpd), ensuring that a full cycle fits within the central region. The sine wave was presented with a fixed phase offset of 0, and the boundary between the center and surround regions was sharply defined. The larger (surround) circle had a radius of 3°, while the smaller (central) circle measured 0.35° in radius. The human fovea contains a rod-free area of approximately 0.35–0.5° radius (Curcio et al., 1990); although individual differences exist, a 0.7° stimulus remains largely within this zone. This allows for perceptual filling-in of the foveal region by surrounding information when stimulating rods (Fig. 2A). The orientation of the gratings in the center and surround could be either parallel (continuous stimulus) or orthogonal (discontinuous stimulus), with the central grating presented either horizontally or vertically (Fig. 2B). Due to the sharp edge between the center and the surround in the discontinuous stimulus, the difference between discontinuous and continuous stimuli was not limited to the spatial frequency of the gratings of 1.4 cpd, but extended to a range of spatial frequencies, with a major peak at about 1.2 cpd and a full-width-at-half-height of about 1.8 cpd. Under photopic and scotopic viewing conditions, stimulus contrast was set to 1 relative to the neutral background. Under mesopic conditions, rod-mediated stimuli were presented at maximal contrast (0.386), while L-cone-isolating stimuli were shown at a reduced contrast of 0.138. L-cones were selectively stimulated under mesopic conditions to achieve higher effective contrast, as stimulating all cone types (while silencing rods) would have produced lower contrast.

With four independent primaries, we could independently stimulate the three cone types and rods under mesopic conditions. However, there is also a fifth type of photosensitive cell, which could not be controlled independently: intrinsically photosensitive retinal ganglion cells (ipRGCs) with the photopigment Melanopsin (for reviews see Barrionuevo, Sandoval Salinas, & Fanchini, 2024; Schmidt, Chen, & Hattar, 2011). Although we were not able to explicitly silence ipRGCs, it is unlikely that ipRGCs were activated by our stimulus, because of two reasons: First, the spatial frequency of our stimulus was with 1,4 cpd above the spatial frequency limit of melanopsin at about 0.5 cpd (Nugent & Zele, 2024). Second, other studies found no melanopsin contribution at the background luminance in the mesopic condition of about 20 photopic Trolands (Zele, Adhikari, Cao, & Feigl, 2019).

**Fig. 3:**
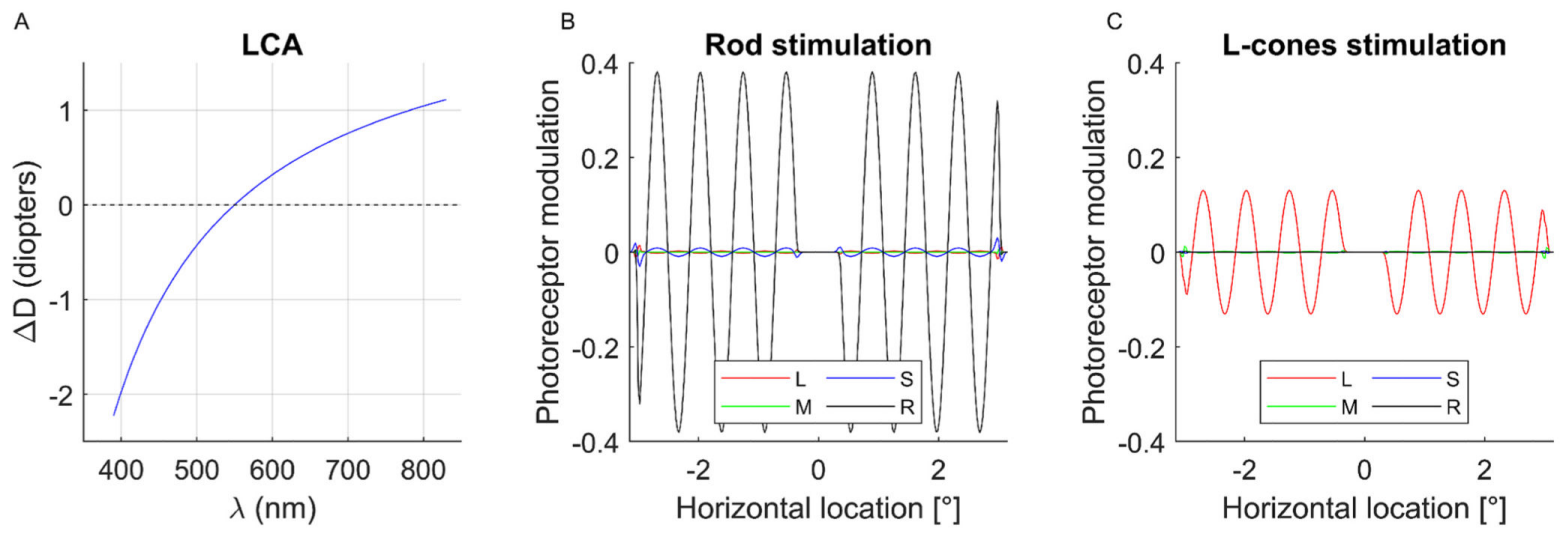
Effects of longitudinal chromatic aberration (LCA) on stimulus contrast. A) LCA expressed as defocus relative to a reference focus at 550 nm. B) Predicted photoreceptor-contrast across horizontal position for the rod-isolating stimulus after applying wavelength-dependent PSFs that incorporate LCA. Rod contrast [R] was preserved (0.386 intended/0.387 achieved), while cone intrusion remains small (L-cone [L]: 0.000/0.034, M−cone [M]: 0.000/0.020, S-cone [S]: 0.000/0.046). C) Predicted photoreceptor-contrast across horizontal position for the L-cone-isolating stimulus after applying wavelength-dependent PSFs that incorporate LCA. L-cone contrast [L] was preserved (0.138 intended/0.131 achieved), with negligible modulation in other photoreceptors (M−cone [M]: 0.000/0.002, S-cone [S]: 0.000/0.000, Rods [R]: 0.000/0.000).

To assess the impact of longitudinal chromatic aberration (LCA) under our mesopic conditions, we modeled wavelength-dependent defocus using a smooth approximation to the empirical data of Thibos, Ye, Zhang, & Bradley (1992), assuming the eye is in focus at 550 nm, which is appropriate for our mesopic background. This model predicts approximately − 1.7 D of defocus at 390 nm and + 0.8 D at 830 nm (Fig. 3A), consistent with human values. To quantify how LCA affects the stimulus on the retina, we generated wavelength-specific point spread functions (PSFs) for a 6-mm pupil that incorporated the predicted defocus at each wavelength. Convolving these PSFs with the spectral stimulus yielded the predicted retinal image under LCA (following Watson, 2015). Across both the full stimulus and the task-relevant stripe region, rod contrast was well preserved for the rod-isolating condition (39% intended, 39% achieved), while cone leakage remained very small (approximately 2–5%; Fig. 3B). For the L-cone–isolating stimulus, L-cone contrast was likewise preserved (14% intended, 13% achieved), and the leakage of the other photoreceptors remained negligible (approximately 0.3–2.6%, Fig. 3C). Thus, LCA introduces only modest wavelength-dependent blur in our setup and does not compromise photoreceptor isolation for the continuity-discrimination task.

### 2.5 Procedure

**Fig. 4:**
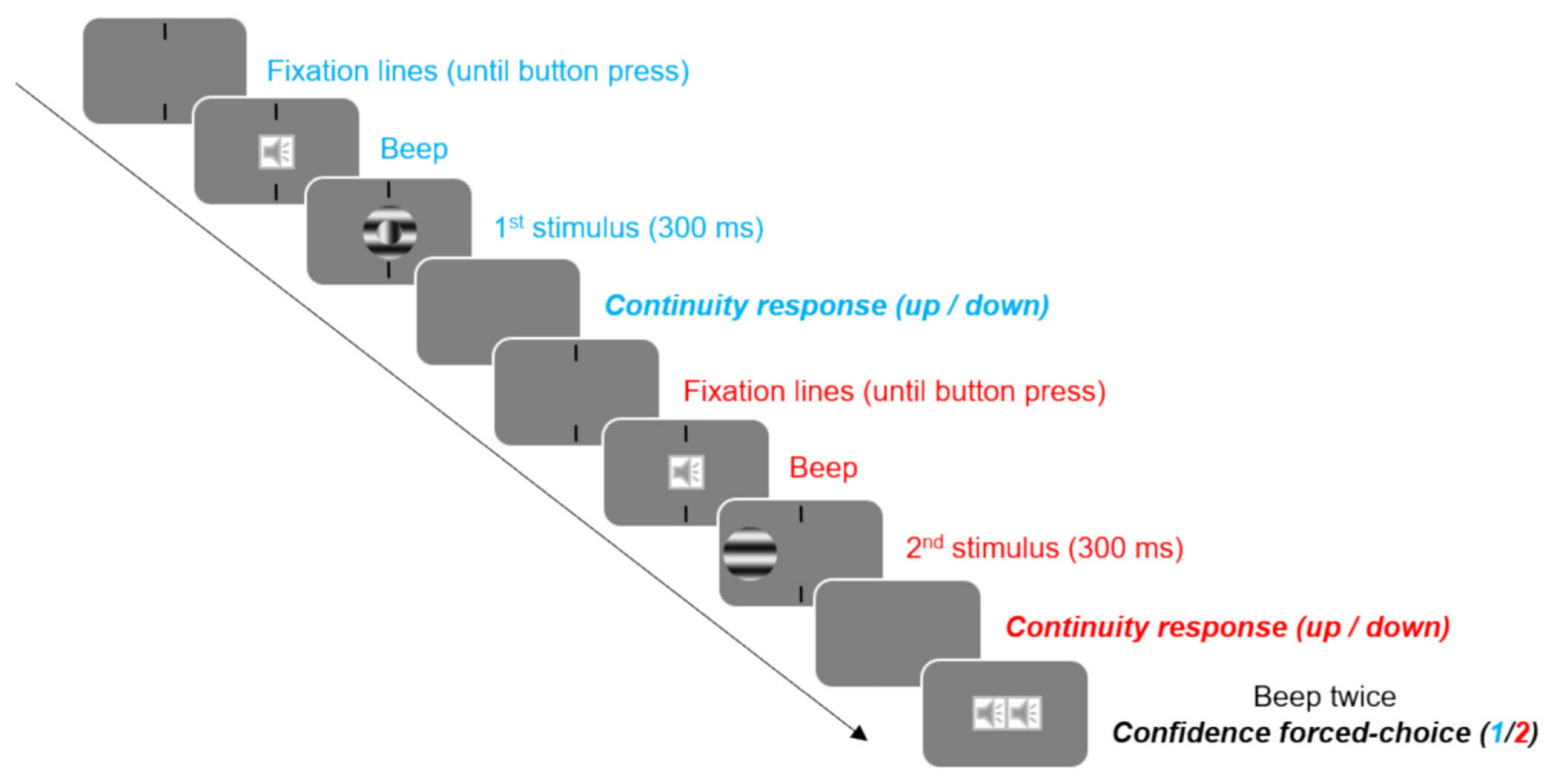
Experimental procedure. Participants reported the continuity of the stimulus at the end of each interval. After responding to the second interval, they indicated which of the two responses they felt more confident about.

Two vertical lines, aligned and separated by an 8° gap, were presented at the center of the screen to guide fixation. Participants were instructed to maintain fixation at the center of the gap throughout the stimulus presentation. Each trial began with a press of the space bar, after which a beep signaled the imminent presentation of the first stimulus. The first stimulus was then presented for 300 ms, either at the center of the screen or 8° to the left. After the stimulus presentation, fixation lines disappeared together with the stimulus, and participants were asked to judge the continuity of the stimulus by pressing the up-arrow key for “continuous” or the down-arrow key for “discontinuous”. The fixation lines then reappeared, and participants initiated the second interval by pressing the spacebar again, following the same procedure for the second stimulus. After making the continuity judgment for the second stimulus, a double beep prompted participants to indicate which stimulus they felt more confident about by pressing “1” (first stimulus) or “2” (second stimulus) on the keyboard. There was no time limit for any of the responses (Fig. 4).

Prior to the experiment, participants underwent a 20-minute dark adaptation period before the scotopic condition and an 8-minute adaptation period before the mesopic condition.

### 2.6 Data analysis

Eye fixations were identified using the default algorithm provided by the EyeLink system. Trials in which gaze position exceeded 2° or in which blinks occurred during stimulus presentation were excluded from analysis (on average: 8.58%). Four participants were excluded because this was the case in more than 10% of trials in at least one of the three viewing conditions.

Although the anatomically rod-free zone spans approximately 0.35–0.5° in radius, the transition from rod-free to rod-populated retina increases gradually rather than abruptly at the boundar. Rod density remains low even up to 1–2° eccentricity, and rod contributions to perception are minimal under mesopic lighting and brief stimulus durations (Curcio et al., 1990; Raphael & MacLeod, 2011). Therefore, the 2° fixation threshold was considered conservative for maintaining the effective foveal presentation of the stimulus. Consistent with this, perceptual performance in the fovea under scotopic conditions was close to chance performance of 50%, indicating that participants did not improve performance by relying on peripheral, rod-mediated vision. In addition to trial-wise fixation filtering, several participant-level exclusion criteria were implemented to ensure data integrity.

Two participants were excluded from the analysis due to poor performance in the discrimination task, as their perceptual accuracy fell below 70% in non-ambiguous conditions (i.e., when the stimulus was presented in the periphery in the scotopic condition). Another two participants were excluded from analysis due to unsuccessful rod–cone isolation. This was determined by above-threshold performance (>80% accuracy) in the continuity discrimination task for foveal stimuli under mesopic conditions, where effective isolation should produce near-chance discrimination. Such outcomes likely reflect individual variability not captured by the standard observer model for 32-year-olds used for calibration, which do not account for age-related differences in macular pigment or lens density.

To further assess the reliability of performance across conditions, we conducted a within-subject validation analysis. Specifically, we computed the correlation between each participant’s accuracy in the scotopic rod-mediated (ScoR) and mesopic rod-isolating (MesoR) conditions for centrally presented stimuli. This analysis revealed a significant positive correlation (Spearman’s ρ = 0.500, p = 0.035), indicating that individuals’ performance was consistent across rod-mediated conditions. This internal validation supports the robustness of the measured effects and helps to rule out confounds related to fixation variability or noise.

**Fig. 5:**
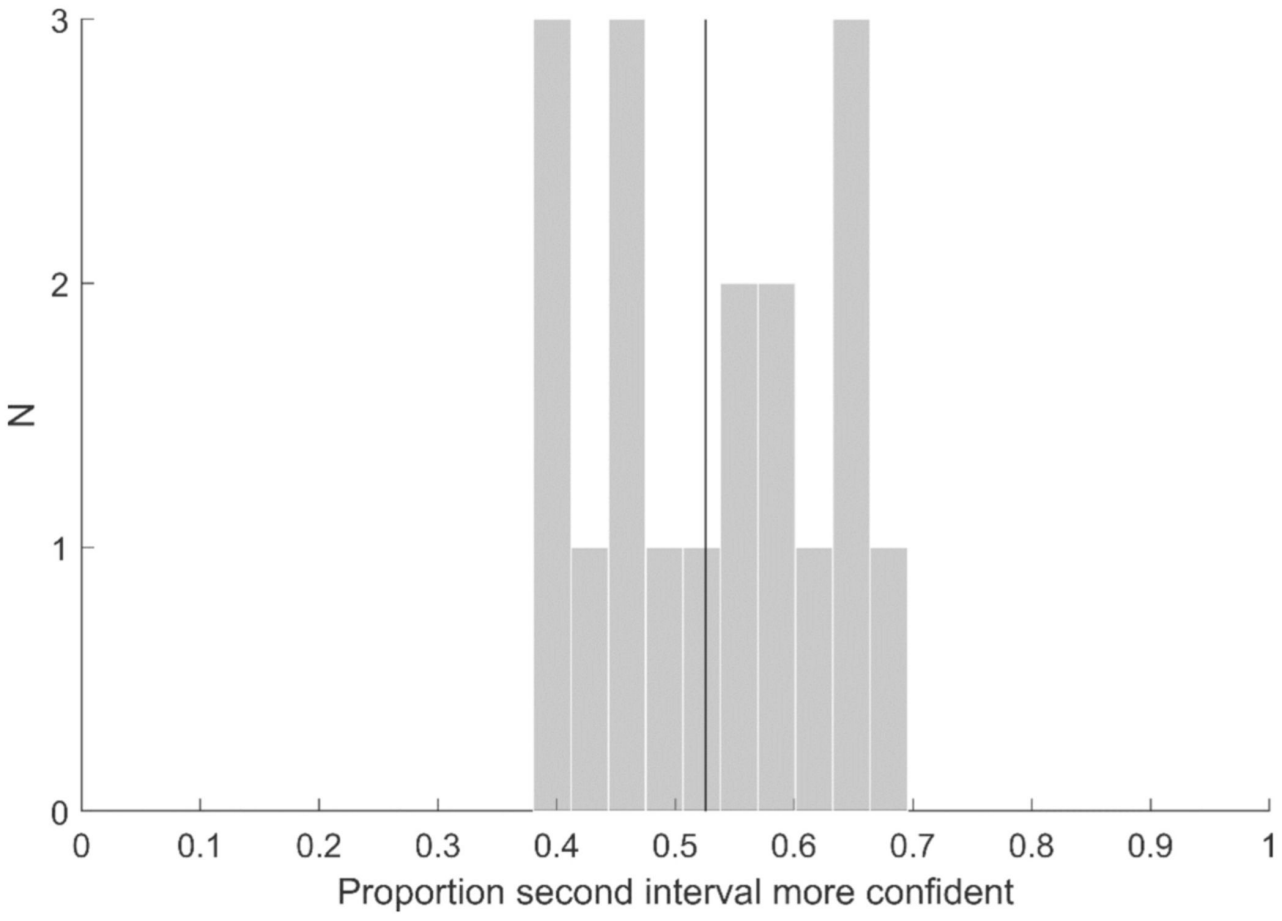
Interval bias. Histogram of the probability of choosing the second interval as more confident. The vertical line represents the grand average across all participants.

For each participant, we analyzed interval bias by assessing the tendency to consistently choose either the first or the second interval as the more confident response. Specifically, we calculated the probability of choosing the second interval as more confident, averaged across all trials and the three conditions. One participant was excluded due to an interval bias exceeding the range [30%, 70%]. Aside from this participant who consistently selected the first interval as more confident, no interval bias favoring either interval (t(17) = 1.080, p = 0.295) was observed for the remaining participants (Fig. 5).

To assess participants’ ability to detect the foveal signal under different viewing conditions, we calculated and analyzed the proportion of correct responses in the continuity discrimination task.

To evaluate participants’ confidence in their decisions, we computed the probability of selecting one of the stimuli as more confident (*p*_*conf*_) in the confidence preference task. A *p*_*conf*_ value below 0.5 indicates greater confidence in the first interval, while a value above 0.5 indicates greater confidence in the second interval. When both stimuli were presented at the same location (either both in the fovea or both in the periphery), *p*_*conf*_ was defined as the probability of choosing the discontinuous stimulus as more confident. When stimuli were presented at different locations, *p*_*conf*_ was defined as the probability of choosing the peripheral stimulus as more confident.

## 3 Results

### 3.1 Continuity discrimination performance

**Fig. 6:**
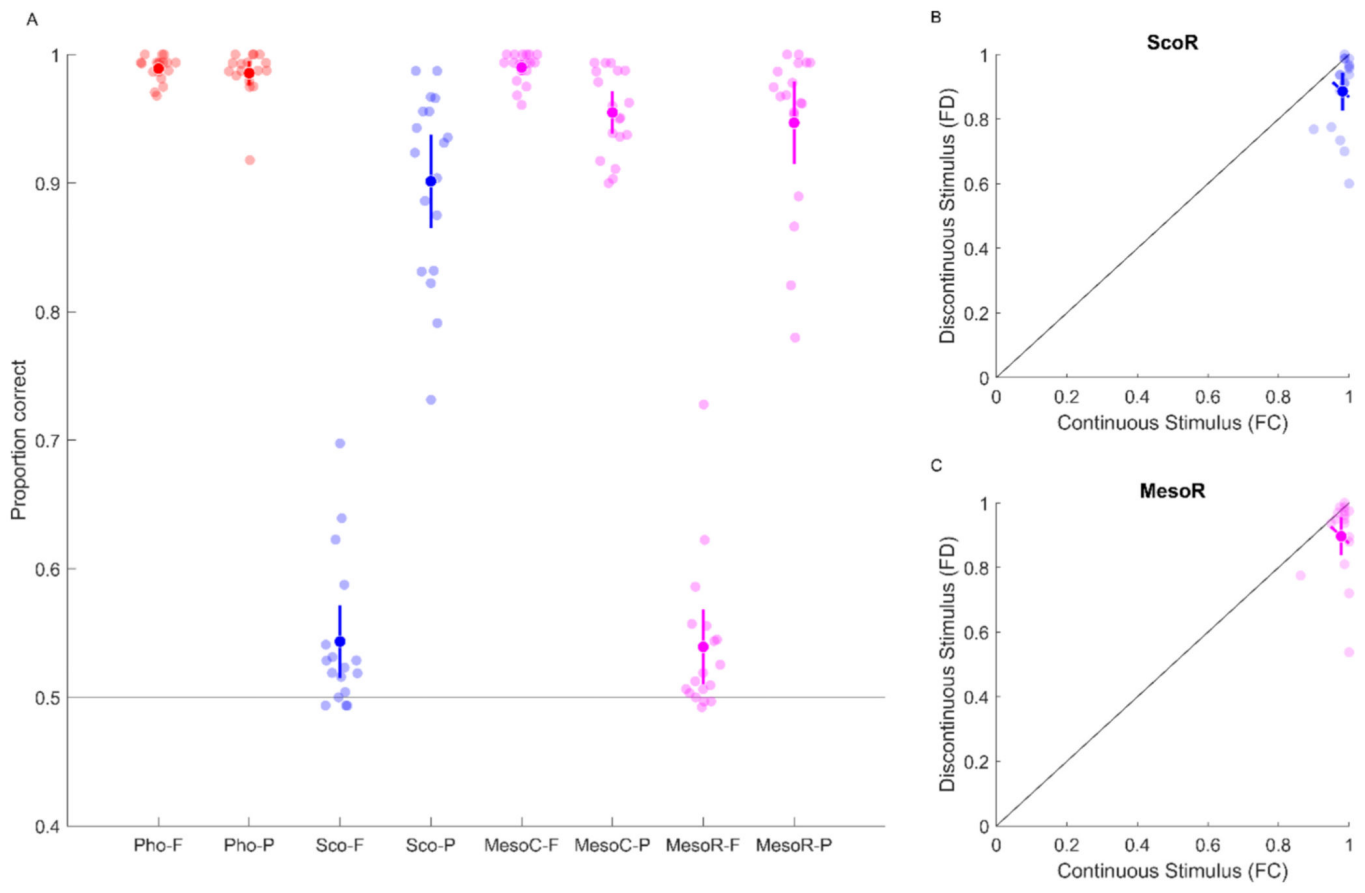
Performance in the continuity discrimination task. A) Proportion of correct responses by condition. Light-colored points represent individual participants; dark points indicate the group mean. Error bars show 95% confidence intervals. B&C) Response probabilities to continuous (FC) and discontinuous (FD) stimuli under rod-mediated conditions (ScoR (top) and MesoR (bottom)) in the fovea. The diagonal error bar represents the confidence interval of the pairwise difference between the x- and y-values and should be evaluated relative to the identity line (Schütz & Gegenfurtner, 2025).

For the continuity discrimination task, we first calculated the proportion of correct responses to assess objective discrimination performance (Fig. 6A).

Under the photopic condition, where vision was mediated solely by cones [PhoC], and under the mesopic condition when L-cones were independently stimulated [MesoC], participants achieved near-perfect performance when the stimulus was presented in the fovea (PhoC: 0.989, 95% CI [0.984, 0.994]; MesoC: 0.990, 95% CI [0.984, 0.996]). Performance was slightly diminished but remained at a high level when the stimulus was presented in the periphery (PhoC: 0.986, 95% CI [0.976, 0.995]; MesoC: 0.955, 95% CI [0.939, 0.971]). Participants were also able to correctly discriminate stimulus continuity when the stimulus was presented in the periphery under the rod-mediated condition in both scotopic (ScoR: 0.901, 95% CI [0.865, 0.938]) and mesopic conditions (MesoR: 0.947, 95% CI [0.915, 0.979]).

When the foveal rod scotoma was invoked by the stimulus, performance decreased almost to chance level (ScoR: 0.543, 95% CI [0.515, 0.572]; MesoR: 0.539, 95% CI [0.510, 0.568]). Analysing the response probabilities (Fig. 6B&C) revealed that, under these conditions, participants predominantly reported the stimulus as continuous—even when a discontinuous stimulus was presented in the fovea (probability of responding “continuous” to a discontinuous stimulus: 0.886, 95% CI [0.827, 0.945] in ScoR; 0.897, 95% CI [0.838, 0.956] in MesoR). This suggests an inability to discriminate the orientation of the center and a bias for continuity reports, possibly due to filling-in of information from the immediate surrounding.

### 3.2 Confidence preference

#### 3.2.1 Confidence preference between continuous and discontinuous stimuli

**Fig. 7:**
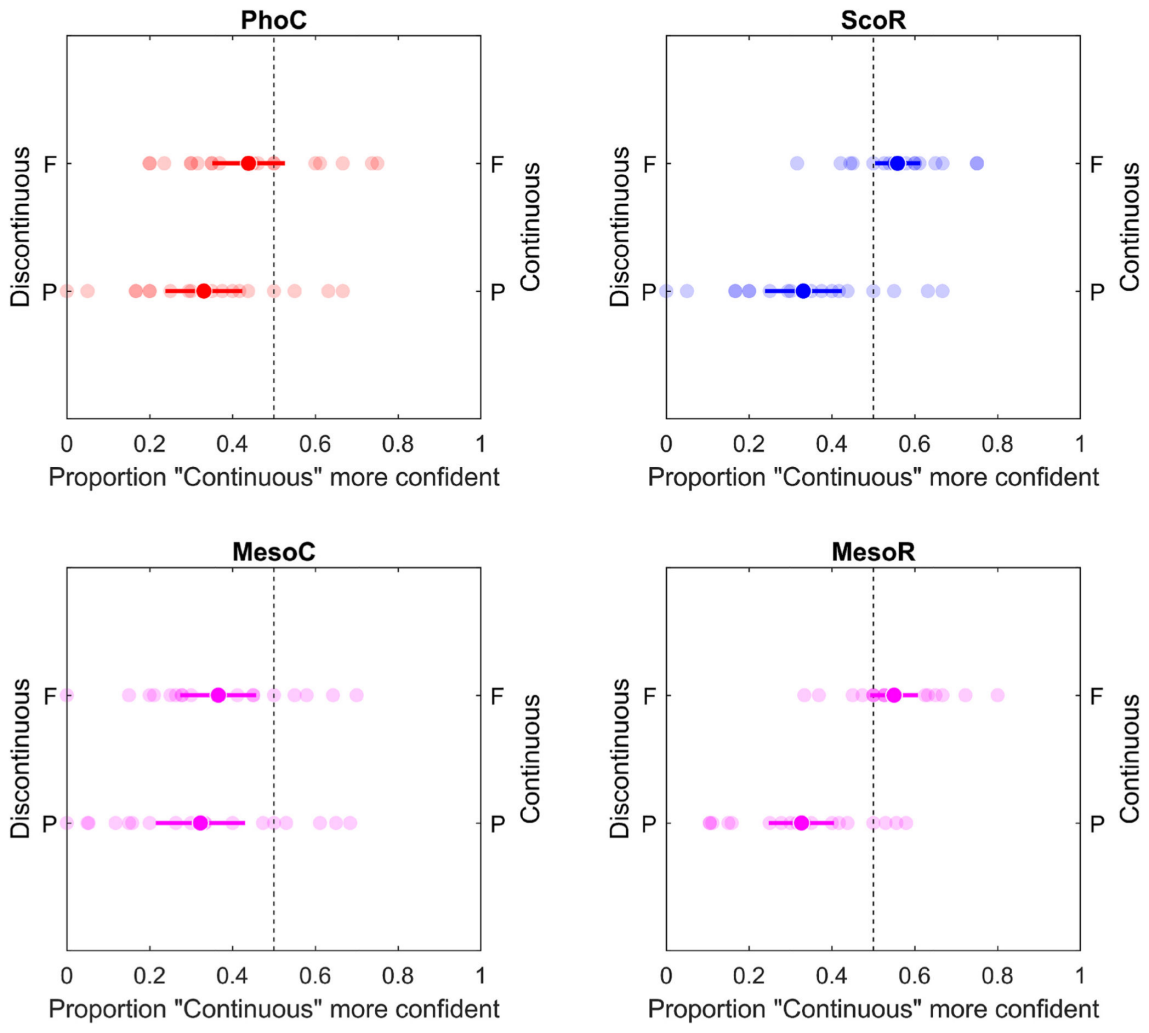
Confidence preference for continuous vs. discontinuous stimuli when both were presented at the same retinal location – either foveal (F) or peripheral (P). The horizontal axis represents the probability of selecting the continuous stimulus as more confident than the discontinuous stimulus. The left y-axis shows conditions where the discontinuous stimulus was presented, and the right y-axis conditions for the continuous stimulus. Within each row (F or P), the two conditions represent comparisons at the same retinal location. Light-colored points represent individual participant averages; dark-colored points indicate the group mean. Error bars represent 95% confidence intervals.

When stimuli were presented at the same location but differed in continuity, a significant confidence bias favoring the discontinuous stimulus (*p*_*conf*_ significantly less than 0.5) was observed in most conditions (PD-PC in PhoC and FD-FC & PD-PC in MesoC, PD-PC in ScoR and MesoR; see Fig. 7).

However, this effect was not evident in cases where the stimulus was presented in the fovea under the photopic condition (FD-FC in PhoC: 0.439 [0.351, 0.527]), as well as under the rod-mediated condition in both scotopic and mesopic vision (FD–FC in ScoR: 0.558 [0.504, 0.613]); FD–FC in MesoR: 0.550 [0.492, 0.608]). This ambiguous confidence pattern in scotopic and mesopic vision further reflects the filling-in effect within the rod scotoma, where the discontinuous stimulus was perceived as continuous despite its physical discontinuity.

#### 3.2.2 Confidence preference between fovea and periphery

**Fig. 8:**
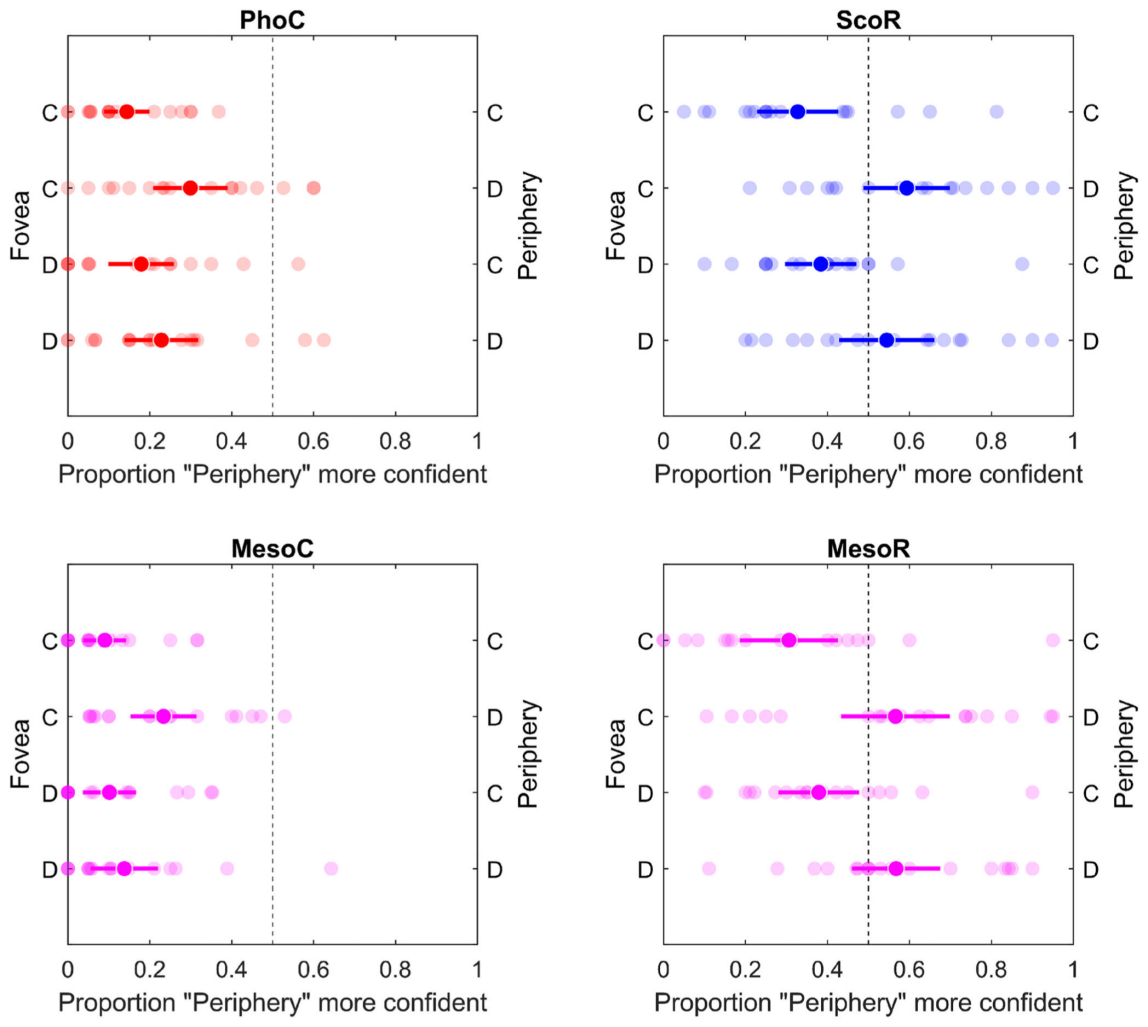
Confidence preference for stimuli with the same or different continuity, presented at different positions – foveal or peripheral. The horizontal axis indicates the probability of selecting the peripheral stimulus as more confident than the foveal stimulus. The left y-axis shows conditions where the stimulus was presented in the fovea, and the right y-axis conditions with the stimulus in the periphery. Within each row (C or D), the comparison was made between the stimuli with either the same continuity (continuous [C] vs. continuous [C], or discontinuous [D] vs. discontinuous [D]) or different continuity (continuous [C] vs. discontinuous [D], or vice versa [D vs. C]). Light-colored points represent individual participant averages; dark-colored points indicate the group mean. Error bars represent 95% confidence intervals.

When comparing confidence preferences between the fovea and periphery for stimuli with differing continuity, participants exhibited a significant confidence bias toward the foveal stimulus (*p*_*conf*_ < 0.5), regardless of stimulus continuity, under cone-mediated conditions (FD-PC & FC-PC in PhoC and MesoC, see Fig. 8).

A significant bias toward the fovea was also observed when the peripheral stimulus was continuous under rod-mediated conditions in both scotopic and mesopic vision (ScoR FD–PC: 0.384 [0.297, 0.471], t(17) = –2.822, p = 0.012; ScoR FC–PC: 0.327 [0.228, 0.427], t(17) = –3.663, p = 0.002; MesoR FD–PC: 0.379 [0.280, 0.477], t(17) = -2.594, p = 0.019; MesoR FC–PC 0.306 [0.186, 0.426],t(17) = -3.145, p = 0.003; see Fig. 8). These findings indicate that participants were unaware that the perceptual information in the fovea was inferred through the filling-in process associated with the rod scotoma. More importantly, despite accurately detecting the veridical stimulus presented in the periphery, participants exhibited overconfidence in the filled-in information at the fovea, revealing a dissociation between perceptual accuracy and meta-cognitive insight.

When the peripheral stimulus was discontinuous, no significant confidence bias was observed between the foveal and peripheral locations (ScoR FD–PD: 0.545 [0.428, 0.661]; ScoR FC–PD: 0.593 [0.488, 0.699]; MesoR FD–PD: 0.567 [0.459, 0.675]; MesoR FC–PD: 0.566 [0.433, 0.698]). This observation further supports the presence of overconfidence in the perceptually filled-in information within the foveal rod scotoma. Despite a statistically significant difference in the performance in the continuity discrimination task between the two retinal locations (t(34) = −22.365, p < 0.001), with mean accuracies of 0.541 [0.519, 0.564] for foveal rod-mediated stimuli (ScoR-F & MesoR-F) and 0.924 [0.896, 0.953] for peripheral rod-mediated stimuli (ScoR-P & MesoR-P), participants’ confidence judgments failed to correspond with this discrepancy and instead were overconfident for the inferred and non-veridical foveal information in the foveal rod scotoma. Confidence preferences for rod-isolating stimuli differed from those for coneisolating stimuli. Continuous and discontinuous rod-isolating stimuli in the periphery under scotopic and mesopic conditions elicited approximately 50% confidence, as discontinuous stimuli create more distinct spatial elements in the periphery than in the fovea due to low contrast and are perceived as more informative. In contrast, continuous rod-isolating stimuli showed a pattern similar to cone-isolating stimuli, exhibiting a stronger bias toward the foveal stimulus.

### 3.3 Summary

Across viewing conditions, continuity discrimination was near ceiling when stimuli were mediated by cones or presented peripherally under rod-mediated conditions. In contrast, when rod-isolating stimuli were presented in the fovea, performance dropped to near chance and responses were strongly biased toward continuity, consistent with perceptual filling-in of the foveal rod scotoma (Fig. 6). Importantly, confidence judgments did not mirror objective performance: participants consistently preferred foveal over peripheral information, even when foveal input was inferred rather than veridical (Fig. 8). This confidence bias was observed under both scotopic and mesopic rod-mediated conditions, indicating that the preference for centrally inferred information persists despite the availability of accurate peripheral input.

## 4 Discussion

We investigated perceptual decision-making under photopic, scotopic, and mesopic viewing conditions. Our results showed that a discontinuous stimulus presented in the fovea was perceived as continuous, not only under the scotopic condition but also under the mesopic condition when the stimulus was exclusively activating rods. This indicates that the foveal rod scotoma is filled-in not only under scotopic conditions (Gloriani & Schütz, 2019) but also under mesopic conditions when light in the fovea could be processed by cones.

Furthermore, consistent with the preference observed when vision was mediated solely by cones under photopic and mesopic conditions, where participants placed greater trust in foveal information, a confidence bias toward foveal vision was also evident under scotopic and rod-mediated mesopic conditions, despite the fact that under these conditions the foveal information was not veridical but instead inferred by the visual system. This rigid preference for foveal vision suggests that humans are unaware of the presence of the foveal rod scotoma under scotopic conditions when only rods are active, and under mesopic conditions involving rod-specific modulation. However, this finding does not necessarily generalize to natural mesopic vision, where rods and cones are modulated simultaneously. The overconfidence in the inferred foveal information might be limited to cases when rod input is selectively driving perception.

Interestingly, we found perceptual filling-in of the foveal rod-scotoma even under mesopic viewing conditions, where cones can be active in the fovea. As rod and cone photoreceptors share downstream pathways to ganglion cells via bipolar and amacrine interneurons, and may even directly couple through gap junctions under mesopic conditions (Bloomfield & Dacheux, 2001; Demb & Singer, 2012; Zele & Cao, 2014), this convergence likely leads to an early loss of information about the specific photoreceptor type, making it difficult for downstream cortical areas to distinguish whether a given signal originated from a rod or a cone photoreceptor. This ambiguity in early visual input may contribute to the brain’s reliance on reconstructive mechanisms, such as perceptual filling-in, to maintain a stable visual experience. Perceptual filling-in is thought to begin early in cortical processing, particularly in V1, where local mechanisms such as receptive field remapping may initiate the process (Weil & Rees, 2011). The completion likely continues in extrastriate areas like V2, V3, and V4, which are more involved in integrating surface features and textures (Komatsu, 2006). In contrast, confidence judgments are typically associated with higher-order cortical regions, including the parietal cortex (Kiani & Shadlen, 2009), prefrontal cortex (Fleming et al., 2010), and striatum (Hebart et al., 2016). The anatomical and functional dissociation between early-stage sensory completion and later-stage metacognitive evaluation may underlie the observed confidence bias: by the time perceptual completion has occurred, information about the original photoreceptor type (rod vs. cone) is no longer available, yet the visual system still produces a seamless percept that is accompanied by high subjective certainty.

The overconfidence for information in the foveal rod scotoma might be related to metacognitive awareness of the spatial resolution across the visual field (Kim & Chong, 2024; Stewart et al., 2020), which can shape internal beliefs or priors about visual reliability. Under photopic conditions, foveal vision offers superior visual acuity due to the high density of cone photoreceptors in the central retina (Curcio et al., 1990), which might lead to a prior belief that foveal input is inherently more reliable. This belief contributes to underconfidence in peripheral vision even when the objective task performance is matched across locations (Toscani et al., 2021). Notably, this prior may persist under scotopic and mesopic conditions, where the advantage of foveal vision is reduced, resulting in an overconfidence in the foveal information that is not supported by actual sensory information. However, this explanation of underconfidence in peripheral vision contrasts with the phenomenon of peripheral inflation, where humans tend to overestimate the amount of detail or information they can perceive in the periphery (Knotts, Odegaard, Lau, & Rosenthal, 2019; Odegaard, Chang, Lau, & Cheung, 2018; Solovey, Graney, & Lau, 2015). This suggests a complex interaction between perceptual limitations and metacognitive bias, where humans may simultaneously be underconfident in their peripheral performance while overconfident in the richness of their peripheral experience. The preference for near-foveal information might not even be inherited from photopic vision if there is a dedicated preferred retinal location (PRL) outside of the rod scotoma that is being used for fixation under low-lighting conditions. A study on patients with central scotomata (Lei & Schuchard, 1997), found that patients use different PRLs depending on the illumination level and in non-human primates, the measured gaze position shifts upwards under scotopic conditions (Barash, Melikyan, Sivakov, & Tauber, 1998; Barton & Sparks, 2001; Goffart, Quinet, Chavane, & Masson, 2006; Spivak, Thier, & Barash, 2014; Spivak, Thier, & Barash, 2021). Hence, it would be possible that the foveal stimulus is preferred over the peripheral stimulus because it is closer to a tentative scotopic PRL. However, it is unclear where such a scotopic PRL would be located. Unlike photopic vision, where visual performance peaks at a single central point that is then used for fixation, scotopic performance increases with increasing eccentricity up to a certain point where it drops off again and that turn-over point depends on the illumination level (Wilkinson, Anderson, Bradley, & Thibos, 2020), resulting in a doughnut-shaped sensitivity pattern rather than a single peak. Furthermore, the eccentricity of that ring might depend on the stimulus size and intensity. For instance, the detection of small stars is best at an eccentricity of 8° (Alexander et al., 2021). Irrespective of whether the preference is inherited from photopic vision or whether it reflects reliance on a nearby scotopic PRL, it does not take into account the fact that there is information missing in the rod scotoma.

The observed overconfidence may also reflect a general preference for filled-in information, akin to that observed in the blind spot, where the absence of photoreceptors resulting from the optic nerve’s connection to the brain, is perceptually filled in by the visual system based on the surrounding information (Ramachandran, 1992). A previous study has demonstrated that humans exhibit overconfidence in the filled-in information within the blind spot compared to physically veridical information (Ehinger, Häusser, Ossandón, & König, 2017). This over-confidence is independent of eccentricity as participants still favor the filled-in stimuli over veridical ones even when both appear at equivalent retinal distances from fixation, which suggests an internal generative bias in the brain — a metacognitive tendency to treat inferred information with high confidence, regardless of its sensory reliability. Perceptual filling-in most likely precedes the estimation of confidence because of two reasons: First, perceptual filling-in can occur as early as V1 and V2 (for a review see Komatsu, 2006), whereas confidence judgments and metacognition are associated with higher-level cortical regions (for reviews see Fleming & Dolan, 2012; Rahnev, 2021). Second, the estimation of confidence corresponds to the evaluation of the quality of a (sensory) decision, based on the underlying signals and the decision criterion (for reviews see Fleming, 2024; Mamassian, 2016). This requires that the inference process itself occurs prior to the evaluation of confidence, though confidence potentially may subsequently reinforce or bias such inferences.

Finally, the observed overconfidence could be related to the effect of internal consistency in decision-making (Caziot & Mamassian, 2021). Rather than relying solely on the fidelity of sensory input, participants may base their confidence on the internal consistency or stability of their perceptual experience over time. A consistent perceptual response tends to yield higher confidence, whereas variability across trials may lead to lower confidence. In the continuity discrimination task, participants most frequently responded “continuous” when stimuli were presented in the fovea. This response consistency may have contributed to over-confidence in foveal vision, where judgments were more stable.

Overconfidence for perceptual completion is not limited to the cases of proximal gaps like the blind spot (Ehinger et al., 2017) and the foveal rod scotoma (Gloriani & Schütz, 2019). Overconfidence for inferred information has also been observed when estimating the number of objects in partially occluded scenes (Men & Schütz, 2024) and for auditory completion in the presence of masking noise (Baykan et al., 2025). Taken together, these findings indicate that the distinction between veridical sensory information and own perceptual inferences is challenging in many scenarios. Whether overconfidence has the same or different causes in these cases remains to be seen.

## Data Availability

Psychophysical data and analysis scripts are available at https://doi.org/10.5281/zenodo.18734668.
